# Apyretic Malarial Menorrhagia

**Published:** 1871-12

**Authors:** J. W. Southworth

**Affiliations:** Toledo


ART. II.—*Apyretic Malarial Menorrhagia. By J. W. Southworth, M. D., Toledo.*

Thinking the following case might be of sufficient interest to publish, from its rarity &c., I take pleasure in sending it in detail:

Miss K. B., aged about 20 years, of a robust and healthy family; parentage Irish, a native of this country, and a resident of Livingston county, N. Y. Temperament, nervous sanguine; applied for treatment of an obstinate and profuse menorrhagia. Said she had been troubled frequently for several years, and had tried several practitioners and did not get much benefit. The present attack had begun five weeks previously, had taken strong cinnamon tea, with some benefit; but it had ceased to have any effect. I found her skin cool; pulse, slow and soft; tongue, slightly coated; bowels, sluggish; appetite, poor; no *pain* anywhere. Believing it to be simple passive menorrhagia, from anemia chiefly, (as she was remarkably so in comparison with the other members of her family,) and also to want of tonicity of the uterine tissues, I gave her F. E. Ergot, gtts. xx, every four hours, with a laxative powder of podophylin, calomel and nitrate of potassa, every second day at bed time. Continued this for two or three weeks, when she reported herself almost well. Discontinued treatment; she was entirely well in about 10 days more. She quit work only one day; I had forgotton to state,

*These lines were written when I learned that Quaglino had published a number of cases of glaucoma cured by simple peripheral incision without iridectomy.

her occupation was that of cooking. In two weeks she had a return of her trouble, but did not apply for treatment until the end of 2nd week, when she was so bad that she had to go to bed. Gave her ergot as before, and ordered cold compresses to the hypogastrium. She was so much better the third day that she got up and went to work, and was well in about eight or nine days more. Remained well two or three weeks and was again attacked; applied immediately for "some more of those drops." Not thinking it best to continue the ergot, I gave her gallic acid, grs. vi. in solution, every four hours, hoping thereby to make the cure more permanent, continued it for twelve days, when she was entirely well, (did not quit work this time.) She now remained amenorrhœic for five weeks, at the end of which time she became menorrhagic, and continued so for two weeks, when she took gallic acid as before, for two days with no benefit. I then resorted to large doses of persulphate of iron in solution *ter die.* Was better the third week, and was almost well at the end of the sixth, when she discontinued the medicine in disgust; she remained nearly well for three or four weeks more, then became suddenly worse. As a forlorn hope she invoked the virtues of the dacoction of cinnamon again, and got some better, continued it for a week longer and was nearly recovered. Remained in that situation for about four weeks more, then got much worse, (did not stop work,) and was quite bad for twelve days, getting better, or rather almost well again ; *without medicine* during the week following the twelve days. At the end of three weeks she got much worse and applied to me. It became evident that there was something unusual in the case, and I expressed my fears to the patient that there might be some polypus or mucous growth in the uterus, of small size, and stated that I did not like to prescribe further until an examination was made. She seemed reluctant to submit to such, and wanted me to give her some more medicine.

Recollecting that I had observed frequent and long drawn sighs, while she was at work, that she had some difficulty in getting to sleep in the forepart of the night, that her tongue was a little tremulous when protruded, I became convinced that nerve debility was a prominent factor in the case. Turning to that excellent work on Nervous Disorders, by C. Hanfield Jones, I found under the head of "Uterine Neuroses" some remarks which confirmed my belief, and I resolved to try the virtues of nerve tonics.

I then gave *ter die*, quinia sulph. grs. ii to iii, and iron by hydrogen, ditto; adding enough podophylin to produce a laxative effect. Also strychnia $\frac{1}{60}$ of a gr. and liquor, potassa arsenitis gtts, iii after meals, To my gratification I found she was much improved in three days. and in nine days was entirely well. I advised her to continue the medicine for a while, but she got tired of taking it at the end of a week. She was amenorrhœic for five or six weeks, then became menorrhagic. At the end of the sixth day of her period, she took five or six of the powders she had left, every four hours and she got well in a week, did not take the solution of strychnine and arsenic; had no pain nor fever. She did not consult me this time. I should mention before going farther, that from the time she got well from taking the quinine and strychnine and arsenic, she improved rapidly in healthy appearance and strength, as well as in ambition. Remarked that she had not been so well in years before. Her menses returned in three weeks from the last attack, and were moderately profuse; but did not cease after the first week. Had a little "show" after three or four weeks, when she got much worse. Was "much worse" for two weeks, when she applied to me for "some more of those powders!" I gave her some two grain powders of quinia sulph. *ter die*. She got worse for two days, and had to go to bed from loss of blood; directed her to take them every three hours. She took them and got no better. Her skin was cool, pulse 76 soft and regular, tongue moist and slightly coated. Had a dull itching pain in the lumbar region for the first time that she recollects. This was Thursday morning. I concluded to try the virtues of larger doses of quinia sulph.; still believing in the theory of nerve debility and the potency of nerve tonics. I gave 4 or 5 grs. of quinia sulph. every three hours until slight cinchonism was induced. Before night she was "ever so much better." This success led me to investigate this matter more closely in order to find the *cause* of this nerve debility The immediate effects of quinine induced me to suspect the influence of malaria. I questioned her regarding the hours of the day when she would be the worst, and to my surprise she stated that she was generally worse in the morning, when she first got up, and that in an hour or so after she had been at work, she would get better; and also that she had noticed that she was generally worse every other day, and that it had been noticable for several years but that she had not spoken to any of her physicians about it, "did not think it made any difference to them if it was so." I was ready to cry "eureka," but I thought I had better *cure my patient* first.

Suffice it to say that on Friday, (the day to be better according to the every other day periodicity which existed previous to taking medicine during this attack,) I gave the same doses as on Thursday, and in the afternoon of Friday, she got up and dressed herself and went down stairs. Saturday she cooked all day, and took two 5 gr. powders that day, and was nearly well.

I continued the antiperiodic doses every other day, with constant improvement until she was entirely well; which was just nine days from the time she applied for treatment. To prevent any more relapes, I directed her to take 18 grains in three doses, every 3d, 4th, 5th, 6th and 7th days from the date of getting well.

On the 3d week from last recovery, she was again menorrhagic; due no doubt to the excitement of getting ready to go out west, and she states she was "just as nervous as could be."

She took antiperiodic doses of quinia sulph. as before and was well in eight or nine days from date of attack.

The absence of all other symptoms of malarial disorder, except the *periodicity* which characterizes this case, is certainly a rare occurrence, especially the absence of fever. However I think its periodicity and prompt amenability to the influence of large doses of quinia sulph alone, is sufficient evidence of its malarial origin.

